# Supplementary figures and images for: Phylogenetic Review of Acaulospora (Diversisporales, Glomeromycota) and the Homoplasic Nature of Its Ornamentations
Source: J Fungi (Basel). 2022 Aug 23;8(9):892. doi: 10.3390/jof8090892 (PMC9502532; doi:10.3390/jof8090892)

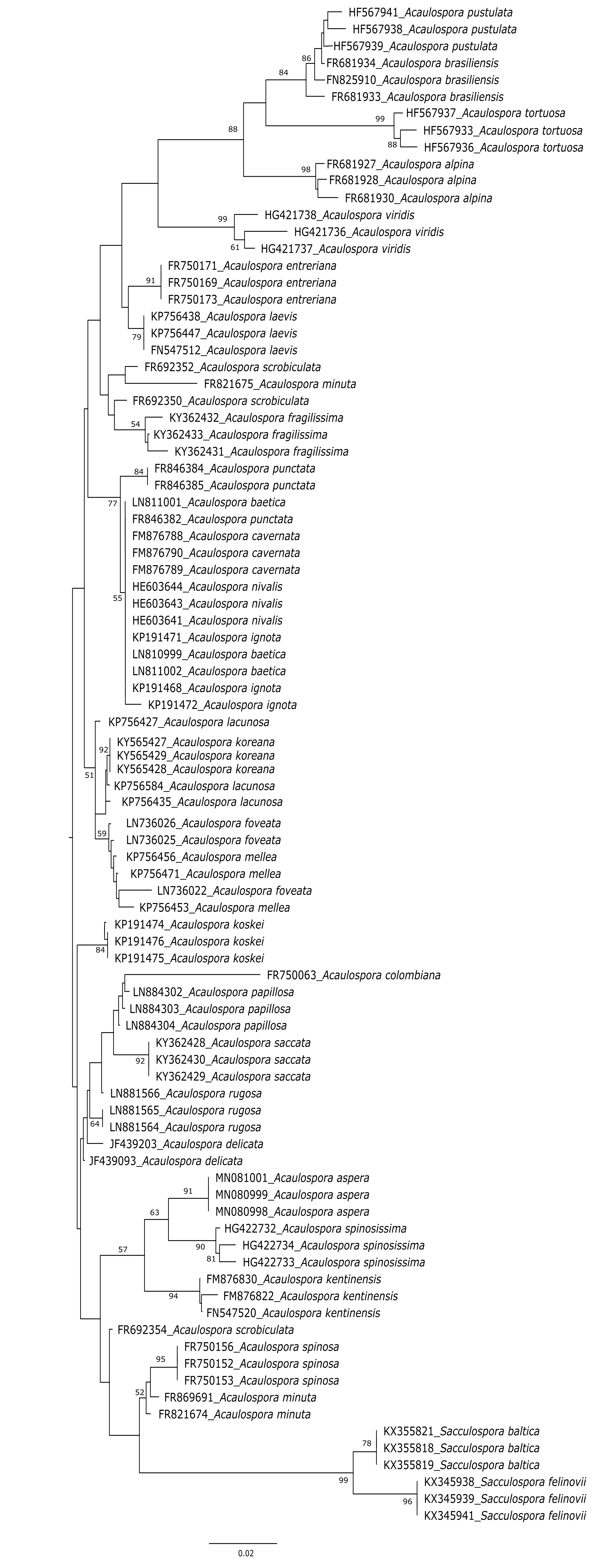

Supplement: Supplementary file 1 [file jof-08-00892-s001.zip › Figure S1.png]

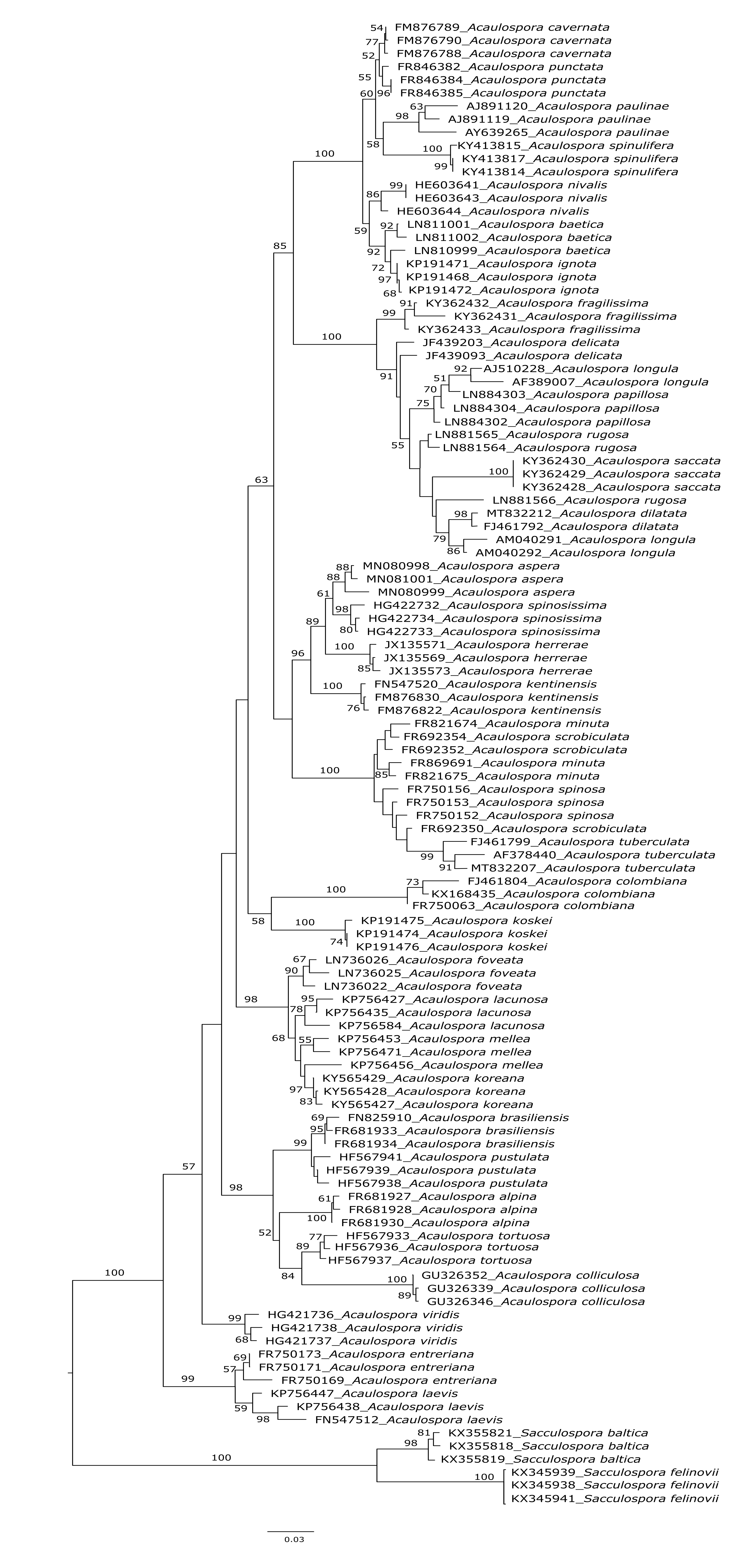

Supplement: Supplementary file 1 [file jof-08-00892-s001.zip › Figure S2.png]
